# 

**Published:** 1871-09

**Authors:** 


### TO OUR SUBSCRIBERS.

It would be too troublesome and expensive to forward bills of subscription in every number of THE COMPANION—we therefore, earnestly ask all of our subscribers who have not paid their subscription, the very small sum of two dollars, to do so now, and you will receive your bill, receipted, in the next number. Friends, don't forget to promptly discharge this part of your duty towards enabling our publisher to furnish THE COMPANION monthly to you and your Professional brethren.

### MEDICAL SOCIETY OF VIRGINIA.

The next annual meeting of the Medical Society of Virginia will convene at Lynchburg on the 18th of October, prox. We have, with great pleasure and gratification, observed the high ethical and educational stand taken by the Profession of the "Old Dominion." They honor themselves by honoring the Profession. In this recognition of the dignity and honor of medicine, we see the highest evidence of the elevated Professional status of our noble brethren of Virginia. We trust all their efforts to advance the interests of the Profession in their State, may be entirely successful. Let the good leaven spread to surrounding States, until, with one voice, the entire Profession, organized upon ethical principles, may demand the recognition of its just claims, by Legislative Bodies, as well as regain the respect and confidence of the people. The solution of this problem rest solely with the Profession. Make the Profession honorable. Bar the door to easy admission within its folds. Let the people see that educational, moral, and scientific merit, can alone find recognition in the brotherhood. Cheap diplomas, issued by inferior medical schools (so-called) and inadequate pupilage, can never give tone to, or confer respectibility upon, the Profession. We are glad to know that the Richmond Medical College will co-operate with the Virginia Medical Society in raising the standard of medical education. It is the duty of every member of the Profession to unite, heart and hand, in the noble work of restoring the old land-marks—in placing the seal of condemnation upon every irregularity the in Profession, and by extending honor to every Institution, and every gentleman, whether as professor, editor or writer, who, for the sake of the Profession, does not *fear* to do right even under the scorpion sting of slander or vituperation. Assail error and you may expect to uncap pandemonium, but stand firmly to right, nevertherless, and the victory will come, all glorious and bright, to the champions of truth.

It may become necessary, in the far distant future, we hope, in order to sustain the true *esprit de corps* of the Profession, to re-organize our Medical Bodies, both State and National. Unworthy men have "crept in unawares," and upon one pretext or another, prompted by the evil genius of selfishness, would sweep away any vestige of principle and ethical law.

The lines of individual character should be clearly, boldly drawn. Let this be the shibboleth upon which, alone, the individual shall be entitled to Professional recognition and affiliation.

### BOOKS, PHAMPHLETS, &C.,

*Braithwaite's Retrospect of Practical Medicine and Surgery.* Part LXIII. July. New York. W. A. Townsend, 177, Broadway, Publisher. Price $2 50 a year. Half-Yearly Parts, $1 50.

This invaluable Retrospect has been kindly sent us by the publisher. As usual, it is replete with the cream of the best current Medical Literature. No practitioner should be without it.

*Half-Yearly Compendium of Medical Science.* Parts VII and VIII, January and July 1871, Philadelphia, S. W. Butler, M. D. 115, South Seventh St. Price $3 00 per annum, in advance. Single numbers $2 00.

This Compendium, while faithfully presenting the current medical literature of Europe, is the only Half-Yearly that does justice to American medicine. An abstract is given of every useful and practical article appearing in our home journals. We are pleased to see Dr. W. A. Green's article, on Hypodermic Medication, published, originally, in THE COMPANION, in Part viii, for July. It ought to be on every practitioner's table.

*The Anatomical Remembrancer, or Complete Pocket Anatomist of the Structures of the Human Body.* Third Edition, with Corrections and Additions. By C. E. Isaacs, M. D., Demonstrator of Anatomy in the University of New York. William Wood & Co., No. 27, Great Jones Street, New York, 1871.

The author, in the first preface, says: "The sole object of this little manual is to recall to the mind of the Student in Anatomy the information he has acquired, either by actual dissection or by the perusal of works which profess to treat more fully on the subject." The succeeding editions of the work have been much enlarged by "modern discoveries in Anatomy," and after giving notice of these, in the preface to the last edition, the author hopes it "will be of increased utility to those for whose use it was especially designed." As a "remembrancer," this little volume is valuable, and as complete as the design will admit of. We regret the publisher failed to give the price on the copy sent us.

*Transactions of the Minnesota State Medical Society. Saint Paul Pioneer Printing Company,* 1871.

The volume before us is very interesting and valuable. The transactions show an unusual number of articles of more than ordinary merit. The Profession of Minnesota is, evidently, harmonious. As a result of this, science is promoted and medicine advanced in their midst.

*New Census and Patent Laws.*—We are indebted to Munn & Co., publishers of the Scientific American, New York, for a neat little bound volume of 120 pages, entitled as above. It contains the complete Census of 1870, showing the areas, and the population of the principle cities. Also, the new patent laws in full, with forms, official rules, directions how to obtain patents, Copyrights, regulations for trade-marks, assignments, how to sell patents, etc. Also, a large variety of valuable information relating to water-wheels, steam-engines, and other mechanism, with many useful tables and recipes, 175 diagrams of mechanical movements, etc. We advise everybody to send for it as above. Price 25 cents. A more valuable compendium, for so small a price, has rarely been published.

Will they not place THE COMPANION on their exchange list?

### LITERARY MAGAZINES.

*The Southern Magazine.* Murdoch, Browne & Hill, 166 Baltimore St., Baltimore Md. Price $4 00 a year.

We are indebted to the publishers for the September number of the above excellent magazine. The table of contents presents a large variety of subjects, enriched by literary beauty and purity of style. The contributions are largely from Southern pens. The tone of this magazine is high and pure; the literature is excellent, and it ought, strictly upon its merits, aside from being Southern, receive the support of our people. We place it on our exchange list with great pleasure, in anticipation of the many good things in store for us by its monthly visits. Will not many of our readers share this pleasure with us, by becoming subscribers ? We earnestly hope so.

### NOTICE TO OUR EXCHANGES.

Several of our Exchanges have been coming to us very irregularly. A few, and only a *few*, have *never yet*, done us the favor to place THE COMPANION on the list of their exchanges. We sincerely thank all who have extended the courtesy of an exchange. We will, at all times, do our best to have THE COMPANION forwarded regularly and promptly. Should it fail to reach our friends, we beg them to notify us of the fact. We also, hope to see every Medical Journal published in America, on our table. Wili our friends not *see* that they are forwarded, and thereby, confer a favor upon the editors ?

In our next number we will give a list, with time and place of publication, of all our exchanges *received.*

